# Houston, we have a problem: Coagulation concerns during long‐term spaceflight

**DOI:** 10.1113/EP092740

**Published:** 2025-05-31

**Authors:** Lewis Fall, Damian M. Bailey

**Affiliations:** ^1^ Neurovascular Research Laboratory University of South Wales Pontypridd UK; ^2^ Faculty of Computing, Engineering and Science University of South Wales Pontypridd UK; ^3^ Faculty of Life Sciences and Education University of South Wales Pontypridd UK

In 2020 there was a case of an obstructive left internal jugular venous thrombosis suspected in an astronaut, discovered during an ultrasound examination performed as part of a vascular research study (Auñón‐Chancellor et al., 2020). The interest of the wider scientific community (White & Wenthe, 2023) was piqued due to the reports of International Space Station (ISS) astronauts experiencing jugular venous stasis and retrospective occlusive thrombi (Marshall‐Goebel et al., 2019). The SkyLab studies of the 1970s led to the original, albeit tentative, suggestion that spaceflight may induce a potentially hypercoagulable state (Kimzey et al., 1975), although this was not supported by any documented scientific evidence. With the National Aeronautics and Space Administration (NASA) to begin exploration of the lunar south pole (Peña‐Asensio et al., 2025) with a view to establishing a long‐term human presence on the moon (Seltikova et al., 2024), and an eventual crewed mission to Mars (Chatzitheodoridis et al., 2024), it would seem that as humans take our first steps towards becoming an extraterrestrial species, we are discovering new risks to the men and women that will lead us there.

Virchow's Triad has long been established (Bagot & Arya, 2008) as a method of assessing coagulopathy risk on Earth. It brings together three key contributing factors of thrombosis, namely, a hypercoagulable state, vascular stasis and endothelial dysfunction, to try to explain the cause of thromboembolism. Essentially, a healthy endothelium provides an anti‐platelet, anti‐thrombotic surface and endothelial damage and/or dysfunction denudes the endothelium of its ‘healthy’ properties, exposing the pro‐coagulant milieu of the sub‐endothelium. Low flow, low‐shear conditions favour the adhesion of platelets and monocytes, as well as the infiltration of plasma components such as low‐density lipoprotein cholesterol and fibrinogen, causing the development of a plaque (Brown et al., 2017). The hypercoagulable state is one that is slightly more complex to explain, but suffice it to say that a continuum exists between healthy and haemostatic abnormalities, hypercoagulable states and ‘overtly’ increased clotting in acute thrombosis. Our research suggests that a key disruptor of normal haemostasis and a potential ‘up‐stream’ trigger to a hypercoagulable state has a free radical basis (please add two references to this sentence: doi: 10.1136/jclinpath20‐5202952 and the Fall (2018) paper as already referenced).

Under resting conditions, the vasculature promotes conditions of decreased platelet adhesion and aggregation by maintaining an environment rich in nitric oxide (NO) and prostacyclin (PGI_2_), and these are subject to redox regulation (Lee et al., 1989; Rubanyi & Vanhoutte, 1986; Shatos et al., 1991). As such, free radical‐mediated oxidative stress downregulates the natural attenuation of platelets in the resting vasculature. Once active, platelets secrete a variety of mitogenic factors, growth factors and chemokines to promote the repair of damaged tissues. Chemokines released include several types of growth factors, which stimulate reactive oxygen species (ROS) formation within the vessel wall, predominantly via the activation of vascular nicotinamide adenine dinucleotide phosphate oxidases (NOX) (Görlach, 2005). ROS generation promotes adhesion and activation of platelets and neutrophils as well as smooth muscle cell and endothelial cell proliferation.

More importantly, the secretion products of activated platelets can induce tissue factor expression via activation of NOX, stimulating coagulation's tissue factor pathway (Görlach, 2005). It has been known since the 1990s that free radicals increase endothelial tissue factor mRNA transcription and expression (Golino et al., 1996) and tissue factor binding to activated coagulation factor VII (FVIIa) is the primary step in secondary haemostasis according to the cellular model and essentially acts as a bridge between the vascular endothelium and the haemostatic system. Similar in vitro results have also been discovered with tissue factor mRNA expression, and accumulation of hydrogen peroxide (H_2_O_2_) in vascular smooth muscle (Herkert et al., 2002). The whole biological ‘point’ of primary and secondary haemostasis is to convert the soluble molecule fibrinogen into the insoluble molecule fibrin, thus stabilising an incipient thrombus. Unfortunately, fibrinogen is not immune to oxidation by ROS. It is suggested that oxidised fibrinogen is more readily converted to fibrin, supports the aggregation of platelets better than non‐oxidised fibrinogen, and is less effective at beginning tertiary haemostasis (fibrinolysis) than its non‐oxidised counterpart (Upchurch Jr et al., 1998).

Not biologically ‘content’ with increasing primary and secondary haemostasis while reducing the efficiency of tertiary haemostasis, ROS are also implicated in the downregulation of coagulation inhibition. Oxidation of low density lipoproteins by ROS results in a potent inhibition of tissue factor pathway inhibitor (TFPI) by specific association with TFPI's C‐terminal region (Ohkura et al., 2004). Finally, thrombin is found to be exquisitely sensitive to oxidation by the myeloperoxidase–H_2_O_2_–chloride system, which impacts interaction with the protein C anticoagulant system and with the serpin antithrombin. As such, there exists, it can be hypothesised, an oxidative‐platelet‐coagulation–amplification pathway (Figure 1b), though further research will be required to confirm this. Research from our laboratory (Bailey et al., 2020) has focused on the unique physiological challenges for the astronaut cerebrovasculature posed by microgravity, and elucidated a potential mechanism that integrates the above hypothesis with spaceflight. Our findings suggest that microgravity‐induced alterations in (cerebrovascular) arterial pressure–shear strain bring about a free radical‐mediated reduction in NO bioavailability, collectively termed oxidative–nitrosative stress (OXNOS), that favours a hypercoagulable state, providing spaceflight with all the ‘ingredients’ necessary to complete the three corners of Virchow's Triad (Figure 1a).

**FIGURE 1:**
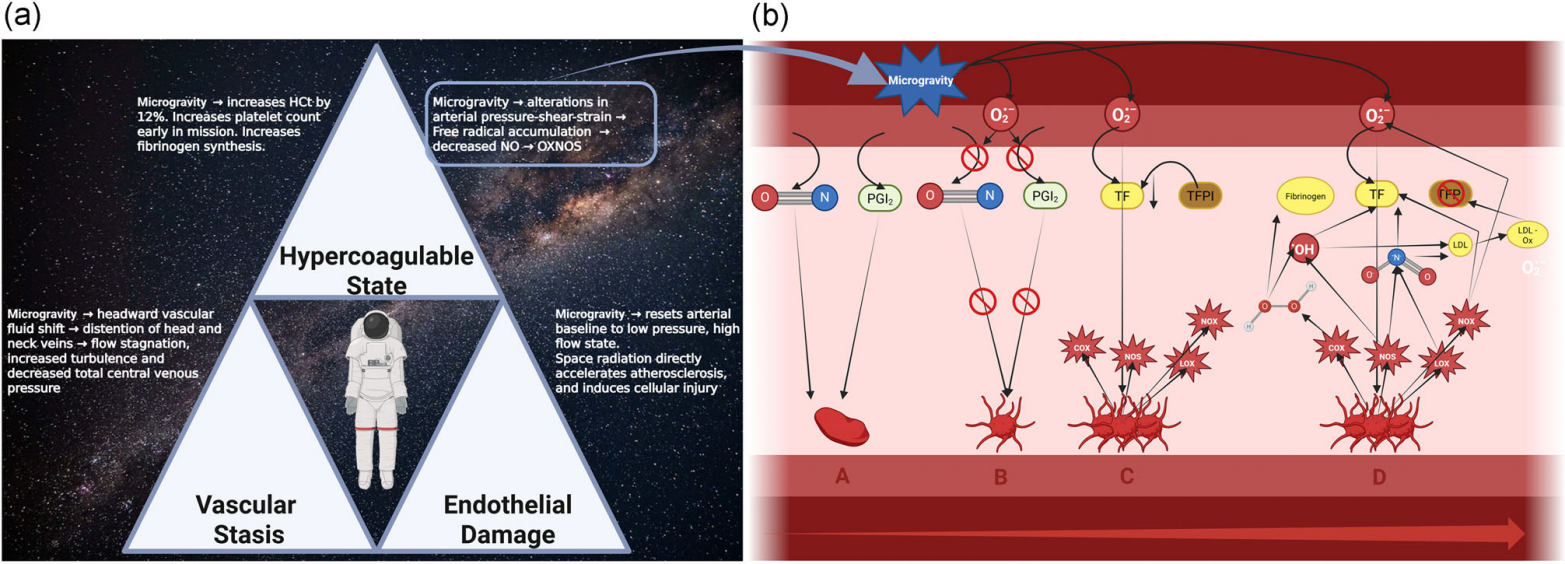
Key concepts of Virchow's Triad as they apply to spaceflight (a; adapted from White and Wenthe, 2023) and our proposed oxidative amplification pathway (b). (a) Resting vasculature promotes downregulation in platelet adhesion and aggregation (Lee et al., 1989; Rubanyi & Vanhoutte, 1986; Shatos et al., 1991). Microgravity induces oxidative stress, which reverses this (Golino et al., 1996; Görlach, 2005). (b) This results in platelets expressing LOX, COX, NOX, NO and NOS, and increases TF mRNA expression (Görlach, 2005). This triggers a chain reaction of increasing TF mRNA expression and downregulation of protein C, and TFPI, potent coagulation inhibitors (Herkert et al., 2002; Ohkura et al., 2004; Upchurch Jr et al., 1998). COX, cyclooxygenases; LOX, lipoxygenases; NOX, NADPH oxidases; NO, nitric oxide; NOS, nitric oxide synthases; OXNOS, oxidative–nitrosative stress; PGI_2_, prostacyclin; TF, tissue factor; TFPI, tissue factor pathway inhibitor.

And a brief point focused on the analytical nuances: given that the in vivo coagulation is such an inherently complex process, there exists an analytical gulf between the measurement of coagulation activation in the acute clinical versus research settings because the routine tests performed (activated partial thromboplastin time, prothrombin time, fibrinogen, etc.) do not fully reflect the in vivo process of coagulation with clear discrepancies between laboratory and point of care analyses (Fowler & Perry, 2015). Near‐patient testing such as thromboelastography and thromboelastometry have bridged this analytical gap (Kvisselgaard et al., 2025) to some extent, but recently our laboratory has applied the method of Fourier transformation haemorheology to determine a single end‐point marker of coagulation activation based on insipient clot fractal dimension. This method was validated in 2014 (Sabra et al., 2014) and used to good effect to detect sensitive alterations in haemostasis in humans (D'Silva et al., 2016; Davies et al., 2016; Fall et al., 2023; Stanford et al., 2015). This method currently requires fresh human blood, delivered to the analyser within 60 s of collection (Figure 2), but has the potential to progress to analysis from vacutainers, which makes it an exciting potential ‘light’ in the current ‘dark’ of spaceflight haemostatic analysis.

**FIGURE 2:**
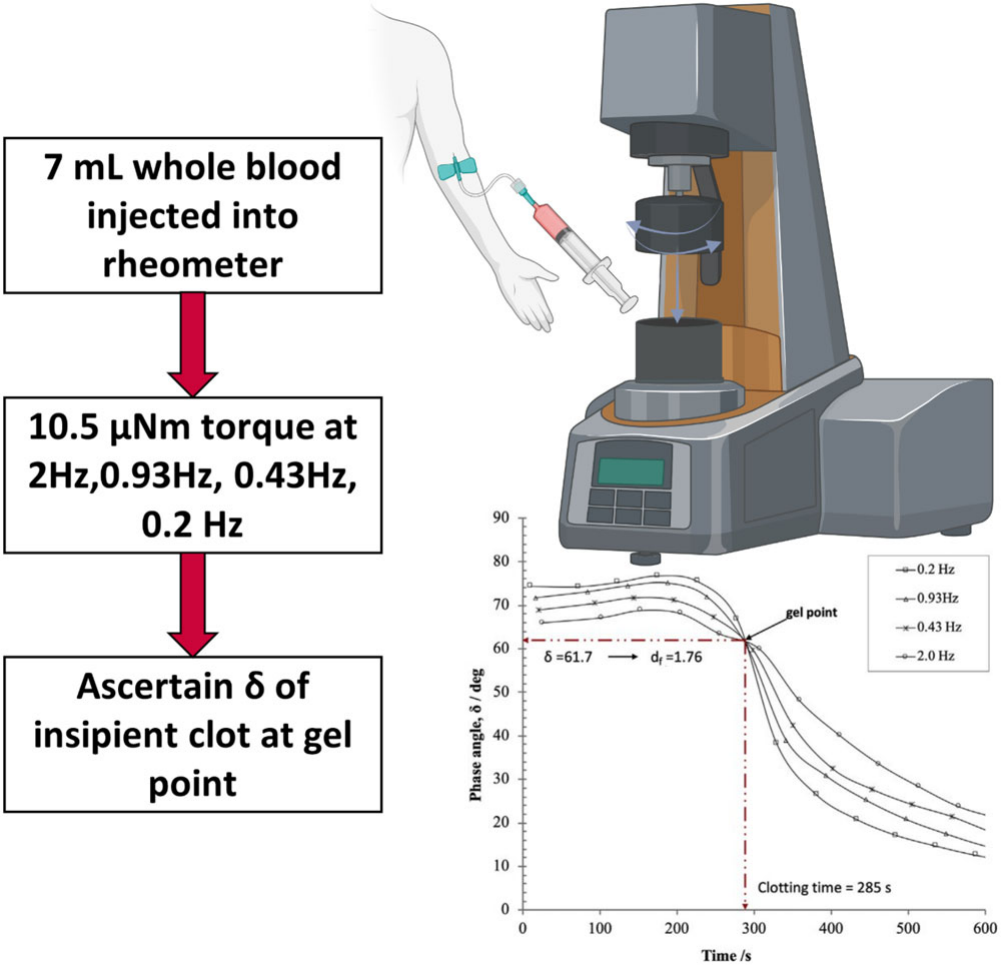
Rheological measurement of coagulating blood: a novel spaceflight biomarker. The graph (Thomas et al., 2020) highlights the phase angle (δ) at four different frequencies plotted as a function of time. The gel point, as detected by a frequency independent δ, indicates the transition from viscoelastic liquid‐like behaviour to viscoelastic solid‐like behaviour. The gel point provides two biomarkers of haemostasis: (i) the clotting time, based on the time to reach the gel point and (ii) the fractal dimension, Df, which can be calculated from the value of δ at the gel point, and provides a ‘one‐stop‐shop’ measure of the structural complexity of the clot.

In closing, we highlight a possible oxidative‐platelet‐coagulation–amplification pathway, driven by oxidative stress that may prove the unifying mechanism to explain the emerging concern of increased thrombotic risk to astronauts. We attest that this is a key mediator in disrupting normal haemostasis on Earth and in space and suggest that future research should consider nutritional (Fall et al., 2018) and/or pharmacological prophylaxis of oxidation as a systemic countermeasure to suppress Virchow's Triad.

## AUTHOR CONTRIBUTIONS

Damian M. Bailey conceived the idea. Lewis Fall and Damian M. Bailey wrote the first draft of the manuscript and revisions thereof. Lewis Fall and Damian M. Bailey approved the final version submitted for publication and agree to be accountable for all aspects of the work in ensuring that questions related to the accuracy or integrity of any part of the work are appropriately investigated and resolved. All persons designated as authors qualify for authorship, and all those who qualify for authorship are listed.

## CONFLICT OF INTEREST

D.M.B. is Editor‐in‐Chief of *Experimental Physiology*, Chair of the Life Sciences Working Group, member of the Human Spaceflight and Exploration Science Advisory Committee to the European Space Agency, member of the Space Exploration Advisory Committee to the UK and Swedish National Space Agencies and member of the National Cardiovascular Network for Wales and South‐East Wales Vascular Network. D.M.B. is also an advisor to Bexorg, Inc. (USA) focused on the technological development of novel biomarkers of cerebral bioenergetic function and structural damage in humans.

## FUNDING INFORMATION

D.M.B. is supported by a Royal Society Wolfson Research Fellowship (Grant No. WM170007).
